# Supplementary material for: Assessment, Treatment, and Follow-Up of Phlebitis Related to Peripheral Venous Catheterisation: A Delphi Study in Spain
Source: Healthcare (Basel). 2024 Feb 1;12(3):378. doi: 10.3390/healthcare12030378 (PMC10855708; doi:10.3390/healthcare12030378)
Supplement: Supplementary file 1 [file healthcare-12-00378-s001.zip › healthcare-2804232-supplementary.pdf]

Delphi reporting guideline (1)

Reporting Criteria	Page Number
I. TITLE AND ABSTRACT: This includes the identification of the study as a Delphi process in the title and abstract, and a structured summary of the study.	P1
II. EPISTEMOLOGY: This refers to the positioning in a specific strand of theory of science (realist or constructivist), as well as the formulation of the objective and the statement of preliminary assumptions, e.g., about possible conflicting perspectives.	P1-2
III. FORMAL CONTEXT: This includes information on the research team conducting the project and other key information on the project background (e.g., funder or objective of the project context, study protocol, ethics committee vote).	P2
IV. KNOWLEDGE BASE: This includes information on the consideration or integration of the current state of research, i.e., the evidence base. It should also be indicated if the designated respondents are aware of it or how it can be ensured that they are aware of necessary contexts or are informed about them and in which social context they are located.	P3-5
V. KNOWLEDGE AND KNOWLEDGE INTEGRATION: This concerns, in particular, the specification of which kind of knowledge stocks and experiences are relevant and which perspectives are necessary to answer the respective research question, as well as how the different knowledge stocks are potentially weighted and how these are to be brought together.	P3-4
VI. DELPHI VARIANT AND ROLE IN RESEARCH PROCESS: Statement and justification of which Delphi variant or which modification has been chosen and how they are situated in the research process.	P3-5
VII. SAMPLE: This includes a comprehensive description of the expert panel with regard to identification, recruitment, knowledge base, sociodemographic data, number of cases (also per round) and, if necessary, recruiting or dealing with refusers or dropouts. The handling of anonymity should also be reported.	P3
VIII. SURVEY INSTRUMENT: The survey instrument must be described in terms of its scope, structure, derivation and testing of questions or items, the ratio of open and closed questions, integrated scale types, and graphic design.	P4
IX. DELPHI ROUNDS: This includes the number of Delphi rounds, as well as disclosure of the termination criterion.	P4
X. FEEDBACK: Disclosure of statistical and graphic representation of feedback (also per round).	P7-9
XI. EVALUATION: This includes the definition of and dealing with consensus and disclosure of the quantitative and qualitative evaluation strategy, and also how these two aspects are weighted and combined.	P3-5
XII. RESULT: The Delphi process (response, procedure, modifications) and the results of the individual Delphi rounds are to be presented. If necessary, reasons should be given why specific elements were changed during the process. The complete procedure should also be presented graphically, e.g., in a flowchart.	P5-9

XIII. QUALITY OF DATA AND INTERPRETATION: This includes reflection on the quality of the data collection and evaluation process. Quality criteria for quantitative or qualitative research should be applied depending on the epistemological positioning and the specific Delphi variant.	P6-9
XIV. DISCUSSION AND LIMITATIONS OF THE FINDINGS: This refers to a critical reflection on the validity claim of the findings, also depending on the epistemological basis.	P9-12
XV. DISSEMINATION: Statement on how the findings will be processed or used beyond the Delphi study.	P12

1. Spranger, J., Homberg, A., Sonnberger, M., Niederberger, M., 2022. Reporting guidelines for Delphi techniques in health sciences: A methodological review. Zeitschrift für Evidenz, Fortbildung und Qualität im Gesundheitswesen 172, 1-11. https://doi.org/10.1016/j.zefq.2022.04.025.
